# A Novel Two-Stage Induced Deep Learning System for Classifying Similar Drugs with Diverse Packaging

**DOI:** 10.3390/s23167275

**Published:** 2023-08-19

**Authors:** Yu-Sin You, Yu-Shiang Lin

**Affiliations:** 1 Professional Master Program in Artificial Intelligence in Medicine, College of Medicine, Taipei Medical University, Taipei 110, Taiwan; g167110006@tmu.edu.tw; 2 Department of Pharmacy, Lotung Poh-Ai Hospital, Yilan 265, Taiwan

**Keywords:** convolutional neural network, deep learning, drug-image classification, induced deep learning, two-stage induced deep learning

## Abstract

Dispensing errors play a crucial role in various medical errors, unfortunately emerging as the third leading cause of death in the United States. This alarming statistic has spurred the World Health Organization (WHO) into action, leading to the initiation of the Medication Without Harm Campaign. The primary objective of this campaign is to prevent dispensing errors from occurring and ensure patient safety. Due to the rapid development of deep learning technology, there has been a significant increase in the development of automatic dispensing systems based on deep learning classification to avoid dispensing errors. However, most previous studies have focused on developing deep learning classification systems for unpackaged pills or drugs with the same type of packaging. However, in the actual dispensing process, thousands of similar drugs with diverse packaging within a healthcare facility greatly increase the risk of dispensing errors. In this study, we proposed a novel two-stage induced deep learning (TSIDL)-based system to classify similar drugs with diverse packaging efficiently. The results demonstrate that the proposed TSIDL method outperforms state-of-the-art CNN models in all classification metrics. It achieved a state-of-the-art classification accuracy of 99.39%. Moreover, this study also demonstrated that the TSIDL method achieved an inference time of only 3.12 ms per image. These results highlight the potential of real-time classification for similar drugs with diverse packaging and their applications in future dispensing systems, which can prevent dispensing errors from occurring and ensure patient safety efficiently.

## 1. Introduction

Medical errors are the third leading cause of death in the United States [1], with medication errors being significant contributors that are largely preventable [2]. The World Health Organization (WHO) estimates that medication errors result in an annual economic loss of USD 42 billion, and they have initiated the Medication Without Harm Campaign. This campaign aims to reduce medication-related global harm by 50% within the next five years [3]. Under current conditions, the lack of uniformity in the appearance of generic drugs poses risks for medical errors [4]. Many existing medications have look-alike/sound-alike (LASA) names, which can lead to potentially fatal clinical issues [5]. Pharmacies have implemented various methods to reduce medication-dispensing errors [6,7]. The Institute for Safe Medication Practices (ISMP) has proposed strategies to mitigate look-alike/sound-alike (LASA) medication errors, such as separate storage locations, color coding, and the use of tall-man lettering [8]. Further evidence, especially from real-life settings, is necessary to support the implementation of safe labeling strategies [9]. Automated dispensing systems allow pharmacists, robotic dispensers, and pharmacy assistants to share the responsibility of medication dispensing, enhancing safety [10]. Automation can only reduce specific errors; workflow adjustments may introduce new errors [11]. Barcode medication administration (BCMA) systems have been promoted to improve medication safety [6]. However, implementing BCMA systems faces technical challenges that must be overcome [12].

In 2016, the National Library of Medicine (NLM) in the United States launched the Pill Image Recognition Challenge to encourage the development of high-quality algorithms for healthcare professionals and the general public to classify unknown medications [13]. A CNN-based system called MobileDeepPill won the first prize in the challenge [14]. Since then, deep learning has been extensively explored for medication classification. Usuyama et al. found that despite its success, the system struggled to differentiate particularly challenging classes, such as pills and capsules, with low-contrast text [15]. Ling S. et al. proposed a multistream approach focusing on challenging medication samples to utilize better domain-specific information in limited-data scenarios [16]. Kwon H.J. et al. proposed a model with a two-step structure for pill-area detection and multiclass pill detection [17]. Patel U. et al. reviewed the relevant literature, highlighting the potential applications of drug classification in pharmaceutical manufacturing and packaging and emphasizing the potential environmental factors that could impact it [18]. Cha K. et al. investigated the influence of illumination sources and backgrounds on medication classification [19]. Recently, Zheng A. et al. applied the Bidirectional Generative Adversarial networks (Bi-GAN) to classify four categories of unpackaged pills by using near-infrared spectroscopy (NIS). This approach effectively addresses common challenges in unpackaged pill classification, including insufficient samples, unbalanced samples, and sensitive identification error costs [20].

In addition to pill recognition, as mentioned earlier, different studies have investigated medication classification in various packaging contexts. Yang et al. explored the application of computer vision in the production process of vial bottles [21]. Nathir A. et al. also studied the impact of illumination sources on image recognition in the production process of ampoules [22]. Liu et al. attempted to classify boxed medications by using image cropping and OCR techniques [23]. Gromova K. et al. combined deep convolutional neural networks (DCNN) with optical character recognition (OCR) and natural language processing (NLP) techniques to extract information from the labels of bottled medication, enabling the retrieval of essential label information from images [24]. Wang J.S. et al. proposed a highlighting deep learning method to classify the blister packaging of medications [25]. Han Y. et al. employed an induced deep learning (IDL) approach to classify medications in blister packaging [26].

Ting et al. conducted a study on blister-packaged drugs [27], collecting 272 different appearances while excluding six types of packaging. They designed a data-augmentation method where each side of each medication was augmented to 72 images: the camera focused on the medication from nine different angles and eight different rotational directions and standardized the input images to 224 × 224 pixels. YOLO v2 was used as the classification model in this study. The F1 score of the backside model (95.99%) exceeded that of the frontside model (93.72%). The study revealed that the backside of the medications typically showed more information, which can improve the classification capability. Certain packaging types exhibited similarities that posed challenges for human visual recognition but could be addressed through a confusion matrix analysis in deep learning classification. Applying these findings is expected to complement the validation process of automated dispensing cabinets (ADCs) or enhance machine assistance to pharmacists.

Han Y. et al. proposed a special IDL framework for medication-image recognition. Image processing was performed to obtain images more conducive to capturing the appearance of medication features [26]. IDL incorporates human experience and cognition into deep learning by performing image processing to enhance classification capabilities, thus reducing the requirement for extensive training data. With this model, they studied the characteristics of diverse groups and adjusted the ROI based on pharmacists’ medication classification experience, allowing the model to classify more detailed features after training at different scales. This approach enables the effective classification of subtle differences in medications.

Previous studies have mainly focused on classifying unpackaged medications or the same type of packaged drugs. Although deep learning methods have been applied to recognize unpackaged drugs with a high accuracy, the proportion of unpackaged drugs is relatively small in clinical operations. Most of them are packaged drugs, and the packages have many different types and characteristics. Although some studies have been conducted to classify packaged drugs based on deep learning methods, these studies have focused on a single type of packaging. However, there are thousands of medications with different packaging types within healthcare institutions used in the clinical-medication-dispensing process, significantly increasing the risk of dispensing errors. Additionally, the similarity in the appearance of generic drugs poses challenges [4]. Therefore, to better meet the needs of healthcare facility users, the practical application scenarios of drugs with diverse packaging need to be explored and studied.

In this study, we proposed a novel two-stage induced deep learning (TSIDL) method to classify diverse-packaging drugs efficiently and accurately. Specifically, the TSIDL method allows the deep learning models to learn the key features of a drug’s image directly based on pharmacists’ dispensing experience. The key features are cropped by an experienced pharmacist from each drug’s image, such as the engraved details on a pill or the text on a box, thus providing deep learning models with more precise content to learn. This special guiding method can result in a higher classification accuracy when classifying drugs.

The first objective of this study is to investigate whether the TSIDL can achieve more accurate classification results than the existing advanced CNNs. In addition, to be practically applicable to clinical operations, the second research objective of this study is to investigate the computational speed of TSIDL. Based on these two research objectives, the first hypothesis is that TSIDL can outperform the existing advanced CNNs regarding various classification metrics. The second hypothesis is that the inference time of the proposed TSIDL method could be slightly slower than other single-stage CNN models since the proposed TSIDL method is based on the two-stage CNN model. However, it is still fast enough to be applied in clinical operations.

Our experimental results show that the proposed TSIDL method significantly improves the performance of classifying visually similar drugs with diverse packaging types. Moreover, we achieved a superior classification accuracy of 99.39% among the 108 diverse-packaging drugs. To our knowledge, this performance represents a state-of-the-art accuracy. This study also demonstrated that the TSIDL method achieved an inference time per image of only 3.12 ms. The result shows the potential for the real-time application of this method in clinical operations.

Our contributions are listed as follows. First, this study represents a pioneering utilization of deep learning to classify similar drugs with diverse packaging. Second, a novel TSIDL method is proposed in this study, significantly enhancing the performance of a CNN-based classifier for classifying similar drugs with diverse packaging types. Third, this study also shows that the TSIDL method achieved an inference time per image of only 3.12 ms. This result highlights the potential for the real-time classification of similar drugs with diverse packaging. In summary, this study demonstrates the proof of concept for the proposed TSIDL in future dispensing systems, which can prevent dispensing errors from occurring and ensure patient safety efficiently.

## 2. Materials and Methods

### 2.1. Materials

#### 2.1.1. Data Resources

**Figure 1:**
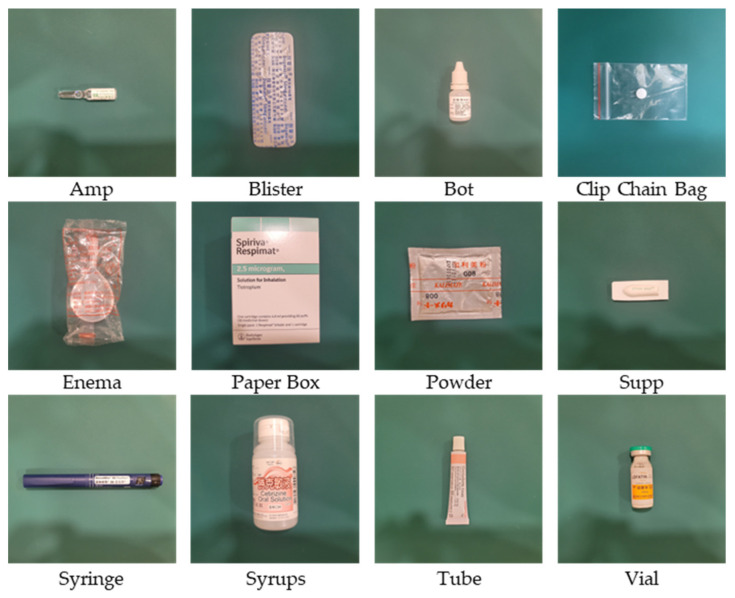
The images illustrate the distinct visual designs of different medication packaging types. These packaging classes demonstrate substantial variations in size, shape, color, text, packaging material, and label markings. Note that Amp denotes ampoule, and Supp denotes suppository.

In this study, we collected medication images following the approved image acquisition request from Lotung Poh-Ai Hospital. The dataset comprised a total of 108 medications with diverse packaging classes, such as 12 types of bottles, five types of suppositories, 18 types of blister packs, and one type of enema. The remaining packaging classes consisted of 9 medications each. Figure 1 illustrates visual representations of the different medication packages, highlighting significant variations in their appearance, such as their size, shape, color, text, packaging material, and label markings.

#### 2.1.2. Data Preparation for Experiments

The data preparation for the experiment was conducted due to the lack of previous research on classifying different packaging variations in medication images. We collected a dataset comprising 108 medication compositions, as outlined in Table 1. During the image-capture process, we simulated the environmental conditions of an actual pharmacist’s work. We did not deliberately control the natural lighting conditions but utilized standard office lighting with a consistent direction and intensity. Similarly, we did not control the environment to reduce shadows and reflections. Moreover, we referred to the data-augmentation method proposed by Ting et al. [27] to augment our dataset. They collected 250 medications with blister-pack packaging and used a camera to capture images of the medications from multiple angles to capture the necessary data. Each medication was augmented 72 times. The camera was strategically positioned at nine different angles to ensure diverse perspectives. Additionally, each image shows the medication from eight different rotational directions, providing comprehensive visual coverage.

**Figure 2:**
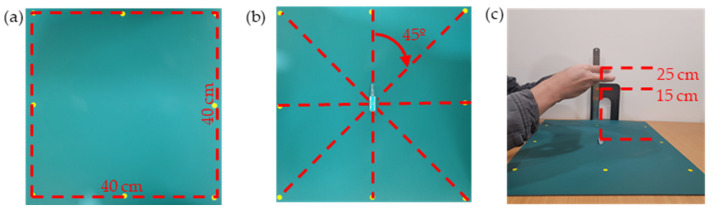
The data-preparation procedure: (**a**) Place yellow dots on the mat at a distance of 20 cm from each dot, creating a square environment measuring 40 cm by 40 cm. (**b**) Determine the position and angle of the medication by aligning with the yellow dots. Then, rotate the medication approximately 45 degrees for each placement. (**c**) Maintain the camera height between 15 and 25 cm, allowing for a range of heights to capture the complete appearance of all medications.

**Figure 3:**
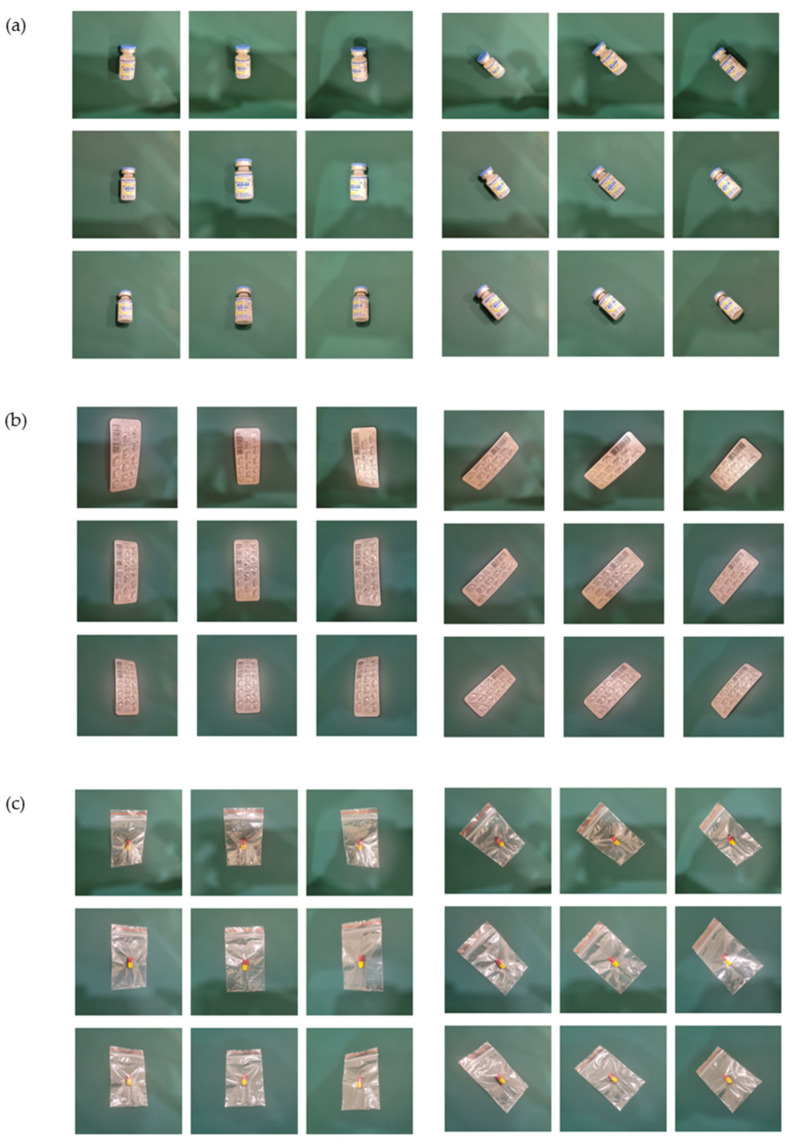
(**a**) Cefa, (**b**) Nebilet, (**c**) Kentamin. We demonstrate the process of capturing medication images with three different packaging types. Firstly, we position the medication at the center of the mat and capture photos from nine different angles, including various tilt and pitch angles. Next, we rotate the medication approximately 45 degrees and capture photos from the same nine camera angles. Finally, we rotated the medication in eight different orientations, completing the collection of 72 images.

Recently, some studies have used smartphones for practical deep-learning-based drug-classification systems and achieved a good performance [19]. Hence, in this study, we capture the drug images by using the main camera of the Samsung Note 20 Ultra smartphone to better match future practical applications. The data collection and augmented methodology in our research are depicted in Figure 2. We utilized a commonly used pharmacist’s desk pad as the background. To control the variation in the rotation angle of the photos of the medications during the augmentation process, we placed a yellow dot on the pad at a distance of 20 cm from each point. Subsequently, we formed a square with dimensions of 40 cm × 40 cm as a reference to align the medications’ angles and centers. By aligning the center of each medication with one of the eight dot centers and aligning the long axis of the medication with the line connecting the yellow dot, we rotated the medication approximately 45 degrees during each image capture. We standardized the tilt and pitch angles during the camera-shooting process, including −20 degrees, 0 degrees, and +20 degrees, resulting in nine different shooting directions. Additionally, we ensured that the angle deviation remained within 2 degrees for each photo. To simulate the operating height of a pharmacist, we used a vertical ruler to calibrate the camera’s height, ranging from 15 cm to 25 cm. This height allowed us to achieve a comfortable handheld height to capture the images. Furthermore, we were able to capture the complete appearance of all the medications. Following the method described by Ting et al. [27], each medication was captured from nine different shooting angles. Additionally, we varied the placement angle of eight different medications at approximately 45 degrees. As a result, 72 different images of each medication could be obtained, as shown in Figure 3. Overall, we obtained a dataset of 7776 images across 108 different medications.

### 2.2. Methods

**Figure 4:**
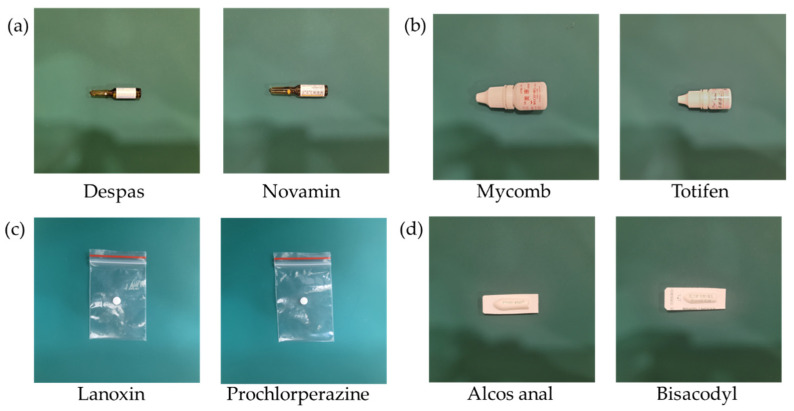
The examples demonstrate similar drugs (SDs) that possess similar features and are prone to be mistaken for one another: (**a**) Despas vs. Novamin: they have a dark-colored ampoule and a white label; (**b**) Mycomb vs. Totifen: they are both white bottles with red letters and packaging; (**c**) Lanoxin vs. Prochlorperazine: they are packaged in clip chain bags and are white, circular pills; (**d**) Alcos anal vs. Bisacodyl: they are packaged in white plastic film with green fonts.

Previous studies have mainly focused on classifying unpackaged pills or medications with the same packaging. However, during the medication-dispensing process, pharmacies encounter various packaging types among nearly a thousand different medications [4]. Moreover, the visual similarities between generic drugs can lead to errors in classification. For instance, Figure 4 illustrates four pairs of medications with similar packaging types, shapes, colors, and other features, resulting in mutual confusion. The examples demonstrate similar drugs (SDs) that possess similar features and are prone to be mistaken for one another: (a) Despas vs. Novamin: they have a dark-colored ampoule and a white label. (b) Mycomb vs. Totifen: they are both white bottles with red letters and packaging. (c) Lanoxin vs. Prochlorperazine: they are packaged in clip chain bags and are white, circular pills. (d) Alcos anal vs. Bisacodyl: they are packaged in white plastic film with green fonts.

Dey M. et al. proposed a two-stage CNN framework to differentiate hand-drawn electrical and electronic circuit components [28]. In the first stage, the circuit components were classified into four distinct groups based on confusion matrices. In the second stage, the circuit components belonging to each group were further classified. Their study employed a two-stage CNN training approach for distinct groups, significantly improving the accuracy from 86.00% to 97.33%. Hence, we introduced a similar two-stage CNN framework to enhance the similarity-of-drug classification.

Some research indicates that drugs’ similarity impacts the classification accuracy of deep learning models. Han, Y. et al. proposed a novel IDL for medication image recognition and significantly improved the accuracy [26]. IDL incorporates human experience and cognition into deep learning, enhancing its ability to recognize medications. Due to IDL demonstrating an excellent drug-classification performance, we adopted the IDL in our approach. We examined the characteristics of different similarity groups (SGs) and set the region of interest (ROI) for medication classification based on pharmacists’ experience. Pharmacists focus on details such as imprints, shapes, and colors when analyzing pills, while for packaging boxes, the overall feature distribution is more relevant. With this understanding, we trained the deep learning model by using different ROIs based on the medication characteristics to enhance its classification accuracy. This approach enables the deep learning model to recognize subtle differences in medications effectively.

**Figure 5:**
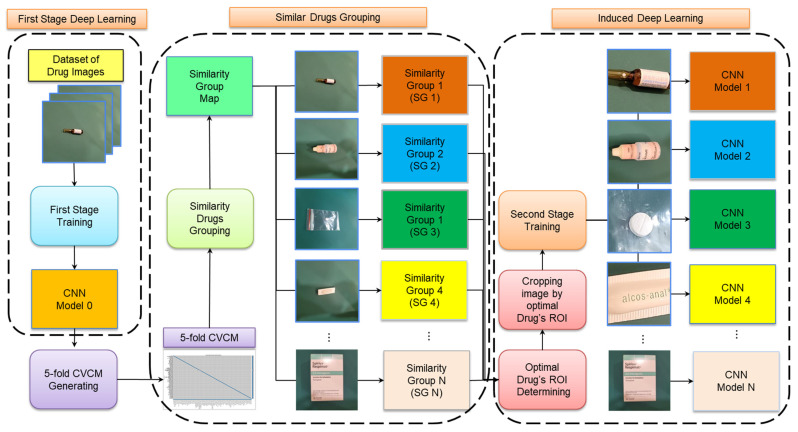
Overview of the proposed TSIDL method: In the first stage, we use the first-stage CNN to train the CNN Model 0 and then use the 5-fold cross-validation method from Model 0 to obtain the 5-fold CVCM. The 5-fold CVCM was subjected to a similar-drugs grouping algorithm to classify SGs, forming N SGs (SG1-N). In the IDL stage, pharmacists utilize their expertise to determine the optimal cropping size for different regions of interest (ROIs) based on the characteristics of each SG. The optimal drug’s ROI images were then used to train Model 1-N in the second stage, corresponding to N SGs.

In summary, the combination of a two-stage CNN with IDL could improve performance in terms of recognizing visually similar packaged medications. Therefore, we introduce a novel framework called two-stage induced deep learning (TSIDL) to enhance the CNN classification performance for similar drugs. In the TSIDL framework, we utilize the first-stage CNN to classify the SGs of medication classes and then employ the second-stage CNN to achieve a more refined classification within each SG for specific medication names. The training framework presented in Figure 5 illustrates the proposed TSIDL approach. In the first stage of deep learning, we train the first-stage CNN by using medication images from 108 different packaging classes to obtain CNN Model 0. Subsequently, we generate a 5-fold cross-validation confusion matrix (5-fold CVCM) by using Model 0. In the similar-drugs grouping stage, we proposed a novel grouping algorithm by using the 5-fold CVCM, creating an SG map that enumerates SG1-N and assigns these drugs to corresponding groups. During the induced deep learning stage, we determine the optimal ROI cropping size based on the characteristics of each SG, guided by pharmacists’ expertise. We utilize different cropping sizes for the images of SG1-N to obtain the best ROIs and then employ the optimal cropped images to train Models 1-N corresponding to SG1-N.

#### 2.2.1. First-Stage Deep Learning

Considering the two-stage CNN structure, the inference time is expected to be approximately twice that of a single-stage method. However, for practical applications in medication classification during pharmacy operations, a fast inference time is required to meet real-time demands. Previous research shows that the AlexNet architecture is known for its simplicity and efficiency [29]. Therefore, we design our two-stage deep learning method based on the AlexNet structure, which should be more readily applicable to practical use. AlexNet, introduced by Alex Krizhevsky in 2012 [30], consists of eight layers, including five convolutional layers and three fully connected layers. We adjusted the output layer of AlexNet to accommodate the classification of 108 different packaging medications in this study. Additionally, to conform with the original design of AlexNet’s input layer, we adjusted the size of the medication images to 227 × 227. In this study, we propose a special CNN architecture based on AlexNet for diverse-packaging drug classification called the single-stage CNN (SSCNN), as shown in Table 2.

#### 2.2.2. Similar Drug Grouping

**Figure 6:**
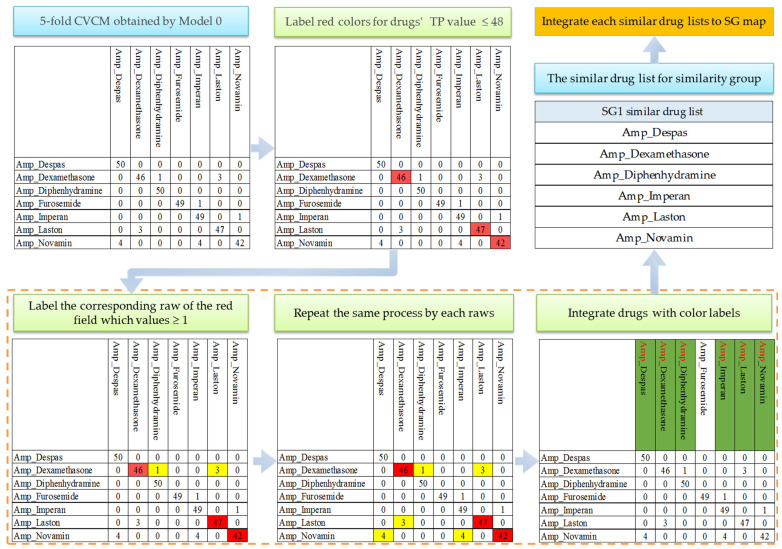
An example of the proposed grouping algorithm for grouping similar medications. Firstly, we constructed a 5-fold CVCM by using Model 0. Then, we examined the true positive values for each medication in the 5-fold CVCM. If the value was less than or equal to 48, we marked that medication red. Next, we checked the false negative values for each medication with a red label. If the value exceeded one or equaled one, we marked those medications in yellow. Subsequently, we grouped the medications with red and yellow labels into an SD list and marked them in green. Then, we could obtain the SD list for the similarity group. We applied the same process for all drug categories. We could obtain the SD lists for SG1-N, and finally, we could integrate them into the SG map.

The confusion matrix (CM) reveals errors in the distribution and patterns. To classify similar medications effectively, we utilized the error presentation of CM. Due to our limited sample size, we conducted a 5-fold cross-validation (5-fold CV) to obtain the 5-fold CVCM. The CM results from each fold were aggregated to generate the 5-fold CVCM [31]. We referred to [27] to group similar medications and designed a grouping process to analyze the 5-fold CVCM, as illustrated in Figure 6. Take AMP as an example to explain the proposed grouping algorithm to group similar medications. Firstly, we examined the 5-fold CVCM generated by the validation set by using Model 0. Then, we checked the true positive (TP) values for each medication in the 5-fold CVCM. When the TP value was less than or equal to 48, we marked the corresponding medication field in red. Next, we checked the false negative values for each medication with a red label. If the value exceeded or equaled 1, we marked those medication fields in yellow. Subsequently, we integrated the medications with red and yellow labels into an SD list and marked them in green. Then, we generated the SD list for SG1. Then, we repeated the process to obtain SD lists for SG1-N, and finally, we could integrate them into the SG map. Notably, the SG map contains multiple drug categories, and the SD list only includes one drug category. For example, this study has four similar groups, AMP, BOT, CCB, and SUPP, which correspond to four SD lists of SG 1–4. The SG map contains these four SD lists.

#### 2.2.3. Induced Deep Learning for Similarity Groups

Y. Han et al. first applied IDL for drug-image recognition [26]. They adjusted the images based on their experience in drug classification. Initially, they deemed the background unnecessary for classifying blister-pack medications and removed it through cropping. Additionally, considering that both sides of the medication provided valuable information for classification, they merged the front and back images after removing the background. This merging process significantly enhanced the classification capability compared to using the original images.

Similarly, in our study, we proposed a novel IDL method to strengthen the classification of medications with various types of packaging. When classifying medications with different packages, we first observe their overall visual features, such as their packaging, size, shape, color, patterns, textures, or text distribution. By doing so, we can effectively classify medications with distinct features, which are easily recognizable. However, in cases of high similarity, we adjust the observation area based on different conditions to obtain more localized details.

**Figure 7:**
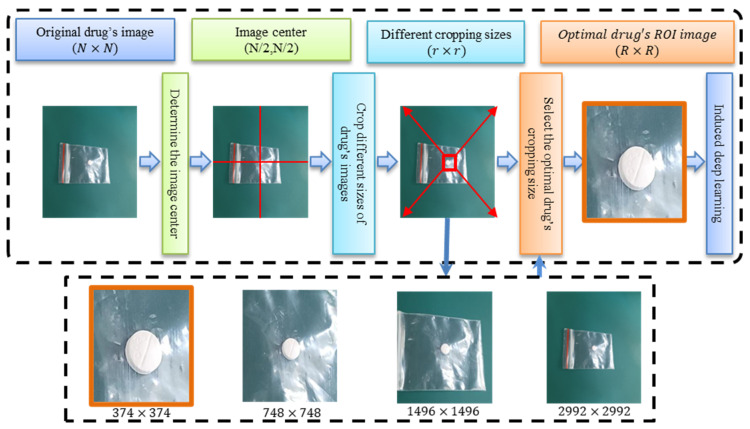
The flowchart of the proposed IDL for SGs is illustrated for pill classification in the CCB class. To capture subtle differences in packaging features, we simulate the steps of a pharmacist carefully examining the medication by adjusting the region of interest (ROI). This lets us discover smaller distinctions within similar drug characteristics, such as texture and imprints. To begin, we load an image of size (N×N) pixels. Then, we position the image center at (N/2, N/2). Next, we expand and crop four windows of different sizes (r×r) from the center. Finally, an experienced pharmacist selects the optimal ROI from the four images of varying sizes, resulting in an image with the best cropping size, sized (R×R). This image with the optimal ROI is then further used within the IDL framework for additional applications.

For example, Figure 7 shows the flowchart for the proposed IDL for the SGs in the CCB class. Our initial focus was on classifying the overall appearance of the medications. We realized that drugs with similar packaging required further examination to determine specific imprints. This approach allowed for a more detailed and accurate classification of drugs within the same SG. Based on the grouping results obtained through the SG map, we enlisted the expertise of a pharmacist with ten years of experience to evaluate the features of each SG. We expand and crop four windows of different sizes (r×r) from the center. This pharmacist used these features to determine the optimal ROI, enabling us to extract critical characteristics such as the color of the label’s text, pill imprints, and the distribution of the packaging text. This method resulted in the generation of optimized models during the second stage of training, leading to an improved performance.

#### 2.2.4. Inference Framework for Proposed TSIDL

**Figure 8:**
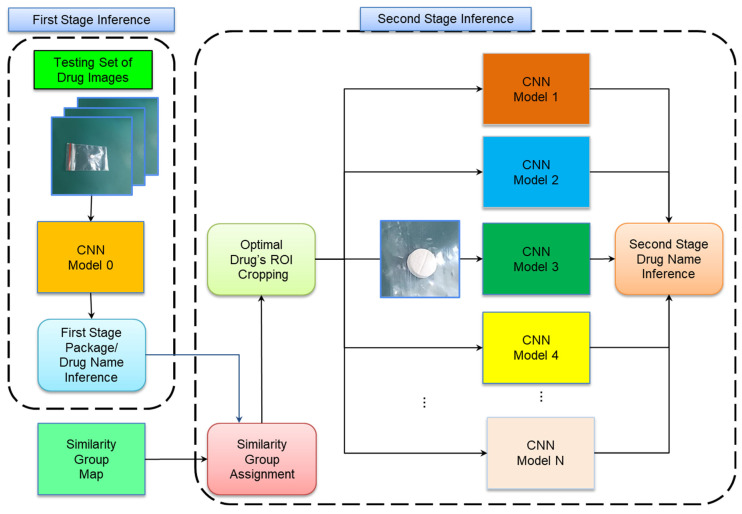
The flowchart of the inference process. In the first stage, CNN Model 0 generates inference results at the drug-name level and the package level. In the second stage, we use the SG map to allocate these drugs to N SGs. Subsequently, the images of SG1-N are cropped by using their respective optimal ROI. Next, each SG image is classified by using the dedicated CNN model corresponding to that SG (CNN Model 1-N), resulting in inference outputs. Finally, the inference outputs from Model 1-N are consolidated to yield the overall inference results of TSIDL.

To demonstrate that the TSIDL method can be applied in real dispensing applications, we designed an inference process for TSIDL. The inference process is depicted in Figure 8. In the first inference stage, CNN Model 0 generates the initial inferences. These inferences assigned the SDs by comparing the inference drug names derived from the SG map. The SG images are then cropped to optimal sizes based on the pharmacist’s determination to enhance their image features. In the second stage, task classification uses dedicated CNN models specific to each SG, resulting in inferences for the corresponding SGs. Finally, the inferences for SG1-N are combined to yield the overall inference results of the TSIDL framework.

## 3. Results

In this study, all the experiments were performed on a 13th generation Intel(R) Core (TM) i5-13500 processor with 64.0 GB of memory and an NVIDIA GeForce RTX 3060 GPU. MATLAB 2023a [32] was used to implement the proposed deep learning architecture. We used a total of 108 different packages of medications. Each medication was augmented to 72 images, forming our dataset for this study. Among them, 40 images were randomly assigned to the training set, 10 images to the validation set, and the remaining 22 images to the testing set. In total, our dataset had 4320 images for training, 1080 images for validation, and 2376 for testing.

In real-world dispensing-system applications, a faster execution time and lower hardware computational costs are required. The five-fold cross-validation can quickly provide reasonable model evaluations with relatively fewer computational resources and a faster execution time. Therefore, this study applies five-fold cross-validation as the model evaluation method. According to the definition of five-fold cross-validation, our dataset was split into five subsets, namely fold 1 to fold 5, to conduct the five-fold CV.

The recall, precision, F1 score, and accuracy were applied as evaluation metrics in this study. Recall quantifies the proportion of correctly classified positive instances. Precision calculates the proportion of true positives among all instances classified as positive. The F1 score is an evaluation metric derived from the combination of recall and precision, providing a balanced measure of the model’s performance. Accuracy shows the overall performance of the models. These values can be calculated based on the following formulas by using the (r × r) CM. Nij represents the number of (i,j) in the CM. True positive (TPi) represents the number of images labeled as class-i that match the inference class. False positive (FPi) represents the number of images labeled as other medications but inferenced as class-i. False negative (FNi) represents the number of images labeled as class-i but inferenced as other medications by the CNN model. Based on these values, we can calculate each class’s recall, precision, F1 score, and accuracy separately. In multiclass classification, the macro-precision (macroP) is the average of the precision values for each class [33]. Similarly, the macro-recall (marcoR) and macro-F1 score are obtained by averaging the individual class metrics. These evaluation metrics are calculated by using the following formulas:

(1) $$macro-recall=\frac{1}{r}\sum_{i=1}^{r}\frac{TPi}{\left(TPi+FNi\right)}$$

(2) $$macro-precision=\frac{1}{r}\sum_{i=1}^{r}\frac{TPi}{\left(TPi+FPi\right)}$$

(3) $$macro-F1\;score=2\times \frac{macroP\times macroR}{\left(macroP+macroR\right)}$$

(4) $$\mathrm{Accuracy}=\sum_{i=1}^{r}TPi/(\sum_{i,j=1}^{r}Nij)$$

### 3.1. Results of First-Stage Deep Learning

In this section, the experimental settings and results will be detailed. MATLAB 2023a [32] was applied to implement the proposed SSCNN architecture. The initial learning rate and batch size were set to 0.0001 and 32, respectively. Stochastic gradient descent with momentum (SGDM) was used to optimize the training process. Additionally, the pretrained model was applied to avoid overfitting. The maximum number of training epochs was set to 100. Notably, the training weights files were automatically saved after each epoch in the training process. Finally, we select the model with the highest validation accuracy as the final model. 

**Figure 9:**
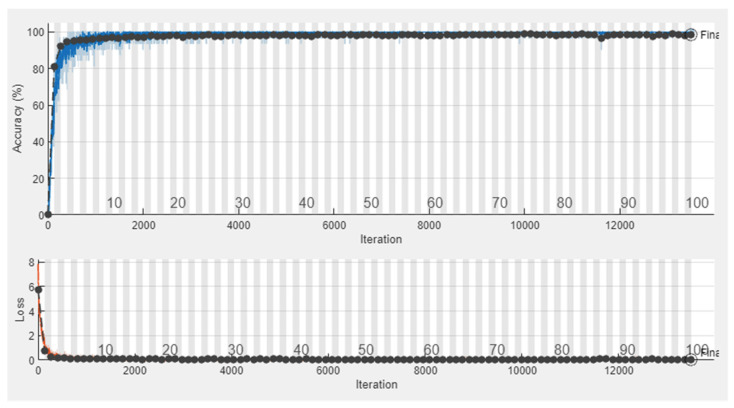
The training process of Model 0. In the upper part of the figure, the blue line represents the training accuracy, and each epoch is denoted by a black dot indicating the recorded validation accuracy. In the lower part, the orange line represents the training loss, and each epoch corresponds to a black dot representing the recorded validation loss.

Figure 9 illustrates the training process of the SSCNN. In the upper part of the figure, the blue line represents the training accuracy, and each epoch is denoted by a black dot indicating the recorded validation accuracy. The best validation accuracy achieved was 98.98%. In the lower part, the orange line represents the training loss, and each epoch corresponds to a black dot representing the recorded validation loss. After 50 epochs, we observed that the validation loss decreased to below 0.13 and then stabilized.

**Figure 10:**
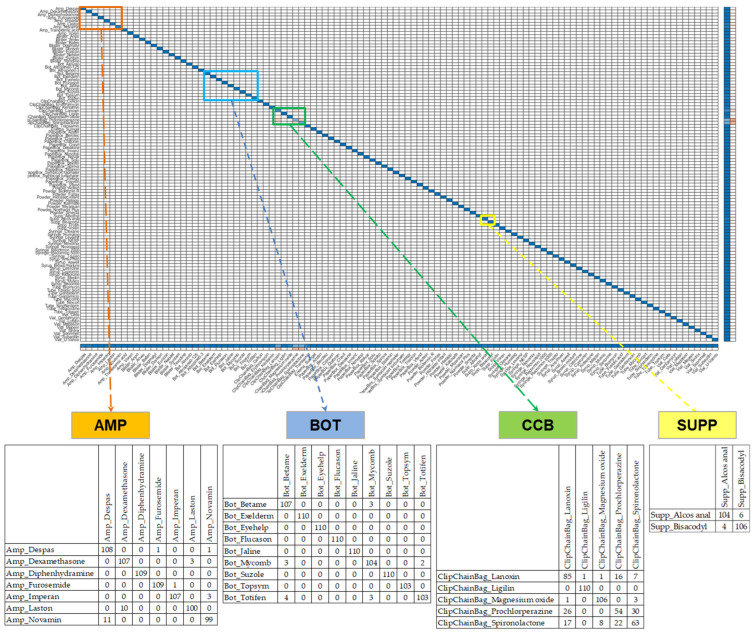
The classification results for drug-name level were obtained from the 5-fold CVCM of the CNN model in the first stage by using the testing set. Upon reviewing the results, it can be observed that significant errors are present in specific packaging classes, namely AMP, BOT, CCB, and SUPP. Particularly, the errors in the CCB classes are particularly pronounced. In the CCB’s 5-fold CVCM, it can be seen that Prochlorperazine has a TP value of only 54, with 26 instances misclassified as Lanoxin and 30 instances mistakenly classified as Spironolactone. Similar situations exist for Spironolactone, with a TP value of only 63, including 17 instances classified as Lanoxin, 8 as Magnesium Oxide, and 22 as Prochlorperazine.

Figure 10 presents a comprehensive summary of the five-fold CV results by using the five-fold CVCM representation for the testing set. Impressively, a drug-name-level accuracy of 98.16% was achieved, indicating the high performance of the proposed SSCNN. However, upon closer examination of the misclassified samples, notable errors were observed at the package level, particularly in the AMP, BOT, CCB, and SUPP classes. Particularly, the errors in the CCB classes are pronounced. In the CCB’s five-fold CVCM, it can be seen that Prochlorperazine has a TP value of only 54, with 26 instances misclassified as Lanoxin and 30 instances mistakenly classified as Spironolactone. Similar situations exist for Spironolactone, with a TP value of only 63, including 17 instances classified as Lanoxin, 8 as Magnesium Oxide, and 22 as Prochlorperazine.

**Figure 11:**
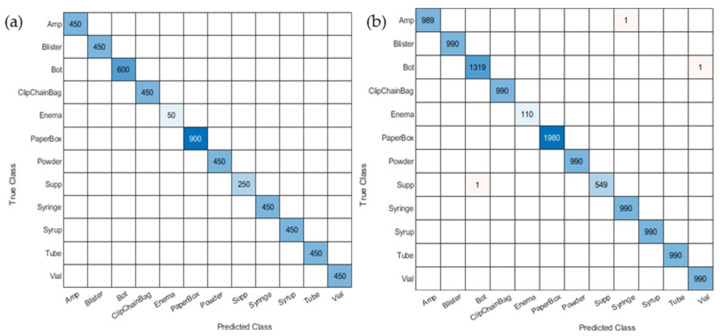
The package-level classification results are as follows: (**a**) the 5-fold CVCM of the validation set demonstrates perfect accuracy; (**b**) the 5-fold CVCM of the testing set exhibits high accuracy in classifying different packages. These 5-fold CVCMs were obtained from the CNN model in the first stage of deep learning. It can be seen that there are some values of 1 in the 5-fold CVCM of the testing set. These represent drugs that are misclassified at the package level.

A dedicated evaluation focusing on the package-level error was conducted, as illustrated in Figure 11, for the five-fold CVCM of the package level. The five-fold CVCM of the validation set demonstrates perfect accuracy. Notably, there are only three misclassifications in the testing set. Specifically, a bot was erroneously classified as vail, an amp was misclassified as a syringe, and a supp was incorrectly labeled as a bot. The remaining errors in the drug names are associated with the same package classes, which are defined as the drug-name-level error.

**Figure 12:**
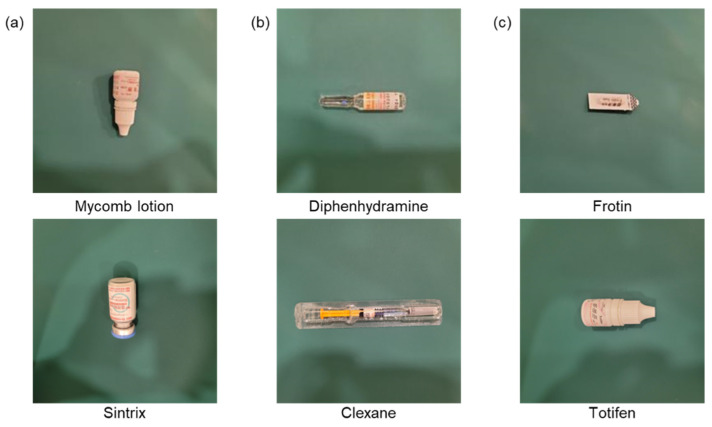
The error classification images at the package level reveal certain similar features despite their differences: (**a**) Mycomb lotion and Sintrix; (**b**) Diphenhydramine and Clexane; (**c**) Frotin and Totifen.

Furthermore, we thoroughly examined misclassified images at the package-level classification, as depicted in Figure 12. Despite visual differences, specific common characteristics were observed within the misclassified package groups. For instance, Mycomb lotion and Sintrix shared the same pink font color, while Diphenhydramine and Clexane exhibited similarities in orange and transparent components. Frotin and Totifen shared features, such as a white color, elongated shape, and black font.

Table 3 presents the classification metrics for the drug-name-level and package-level classification. The accuracy of the drug-name-level classification achieves a 98.16% accuracy. The accuracy of the package surpassed that of the drug names, with all four metrics exceeding a satisfactory value of 99.97%. However, although the drug-name-level classification also achieved above a 98.16% accuracy, it was observed that four classes still exhibited a suboptimal performance, particularly in the case of the CCB package medications. In summary, the package classification demonstrated an overall high accuracy, while errors in the drug-name classification predominantly occurred within groups of similar drugs.

### 3.2. Results of Similar Drug Grouping

**Figure 13:**
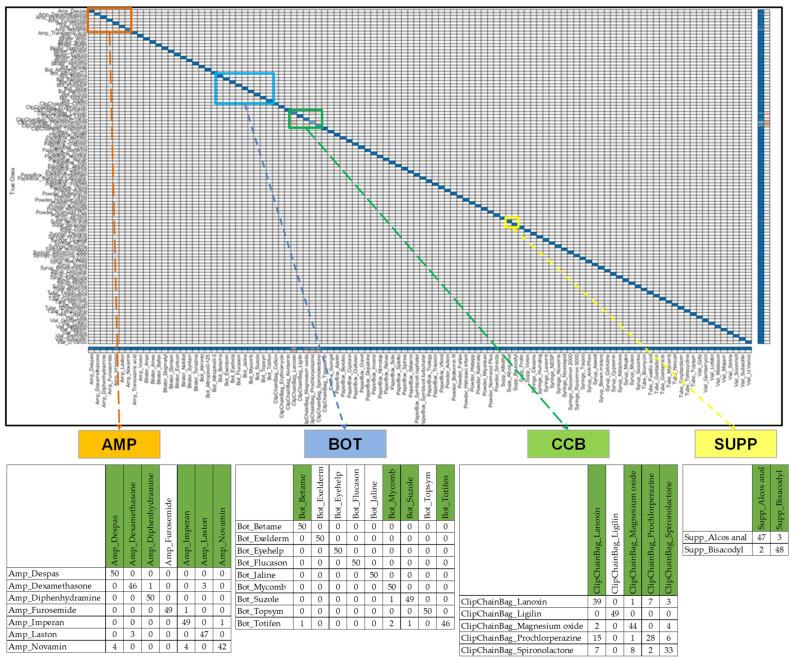
Result of the proposed grouping algorithm. We utilized Model 0 from the first stage to generate a 5-fold CVCM on the validation set. This resulted in the classification of five distinct SGs, comprising six types of AMP (SG1), four types of BOT (SG2), four types of CCB (SG3), two types of SUPP (SG4), and ninety-two types of other classes (SG5), which are labeled green in corresponding fields of 5-fold CVCM.

We obtained a five-fold CVCM by using Model 0 from the first stage, as shown in Figure 13. Through the proposed grouping algorithm, we obtained five distinct SGs (SG1-5), including six types of AMP (SG1), four types of BOT (SG2), four types of CCB (SG3), two types of SUPP (SG4), and ninety-two types of other classes (SG5). The drug images similar to SG1-4 are provided in Table 4. Within SG1-4, there are noticeable similarities in the legends of the same SG, indicating a higher likelihood of classification errors. It is worth noting that the likelihood of classification errors is lower in SG5, as it does not include easily confusable SDs. Therefore, in this study, we focus on discussing the drug features of SG1-4. SG1 comprises amp packaging with similar contours, dark or transparent glass bottles, and relatively small and blurry labels. SG2 exhibits packaging with similar contours and labels with similar-colored blocks. SG3 represents white circular pills in CCB packaging, where the imprints on these pills are not very distinct, and reflections affect readability. SG4 includes packaging with the same contour appearance and green text. This comprehensive examination demonstrates that the grouping algorithm proposed in this study effectively segregates similar drugs. Importantly, we observed that these drugs are consistent with drugs that are prone to be misunderstood by pharmacists.

**Figure 14:**
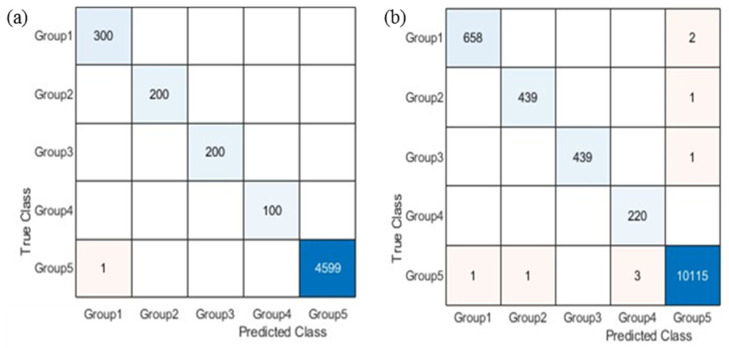
The group-level classification results are (**a**) the 5-fold CVCM for the validation set and (**b**) the 5-fold CVCM for the testing set obtained by using the first-stage CNN. Notably, no classification errors occurred in SG1-4; they only occurred in SG5. It can be seen that there are some values of 1, 2, and 3 in the 5-fold CVCM for the testing set. These represent drugs that are misclassified at the group level.

**Figure 15:**
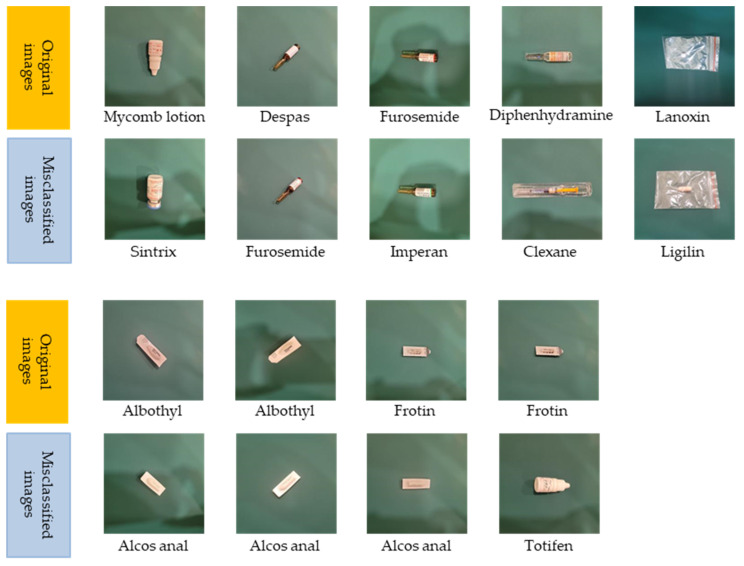
The nine misclassified SD images and misclassified images after grouping, where the drugs have similar appearances, which led to classification errors.

We generated two group-level five-fold CVCMs by using the proposed grouping algorithm on the validation and testing sets, as shown in Figure 14. We observed only one error in the five-fold CVCM generated from the validation set. In the five-fold CVCM generated from the testing set, a total of nine errors occurred. Notably, no classification errors occurred in SG1-4; they only occurred in SG5. This demonstrates that the proposed grouping algorithm is effective at grouping similar drugs (SDs). Figure 15 illustrates the nine misclassified SDs and provides the original images and misclassified images for these SDs.

While our grouping algorithm successfully classed most similar drugs, there were still a few instances where similar drugs were misclassified. Notably, no grouping errors were observed in SG1-4, highlighting the algorithm’s ability to group visually similar packages of SDs accurately. However, we evaluated the accuracy of Model 0 at classifying SGs. As shown in Table 5, the results indicate a group-level recall of 99.84%, a precision of 99.65%, an F1 score of 99.74%, and an accuracy of 99.92%. This demonstrates the strong classification ability of the proposed grouping algorithm for SGs. In summary, our research findings validate the effectiveness of the grouping algorithm at grouping SGs. We employed the proposed grouping algorithm based on similarity and packaging features to group the similar drugs. This methodology enabled us to independently train the models by using each SG (SG1-5). Through this grouping algorithm, we can improve the accuracy of similar drug groups by using the second-stage deep learning approach, which should further enhance the classification accuracy when classifying similar drugs (SDs).

### 3.3. The IDL Effect for Similarity Group

In the proposed IDL workflow, we initially loaded an image with dimensions of 2992 × 2992 pixels. Subsequently, we determined the center of the image at coordinates (1496, 1496). We then extended and cropped four windows of varying sizes from the center, namely 2992 × 2992, 1496 × 1496, 748 × 748, and 374 × 374. Lastly, the selection of the optimal ROI image was guided by an experienced pharmacist from the four different-sized images. This selected image was subsequently subjected to further processing by using IDL.

**Figure 16:**
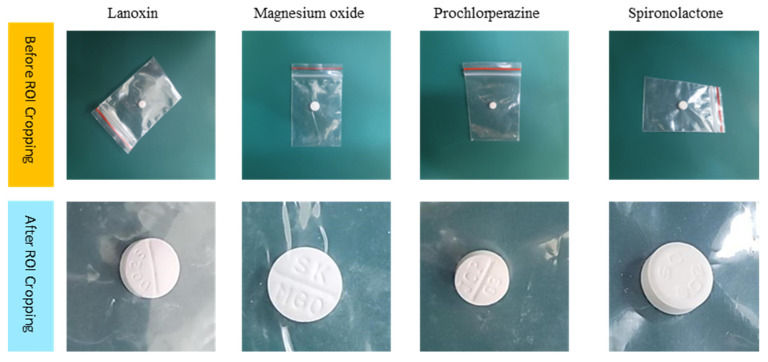
Training image examples of SG3, with the original images in the top row and the region of interest (ROI) images in the bottom row.

This section presents SG3 as an example to illustrate the IDL effect for the similarity group. SG3 comprises four CCB package drugs: Lanoxin, Magnesium Oxide, Prochlorperazine, and Spironolactone. In addition to their packaging, these drugs share common visual characteristics such as being white, round-shaped tablets with imprints and a glossy appearance on the packaging. Following the previously described procedure, we engaged an experienced pharmacist with ten years of experience to select the optimal ROI size of 374 × 374 for the drugs in SG3. Subsequently, we applied this size to crop the training images and applied the second stage of deep learning processing. As illustrated in Figure 16, the image differences in ROI highlight the distinct drug features. Notably, the imprints and textual content on the tablets are not classifiable before ROI cropping. However, after ROI cropping, the imprints and textual content became more prominent. Furthermore, the cropping process also emphasized the more pronounced differences in the pill’s size. In summary, the uncropped images lacked distinguishing features for drug-name classification, whereas the cropped images exhibited more discernible characteristics than the original image.

**Figure 17:**
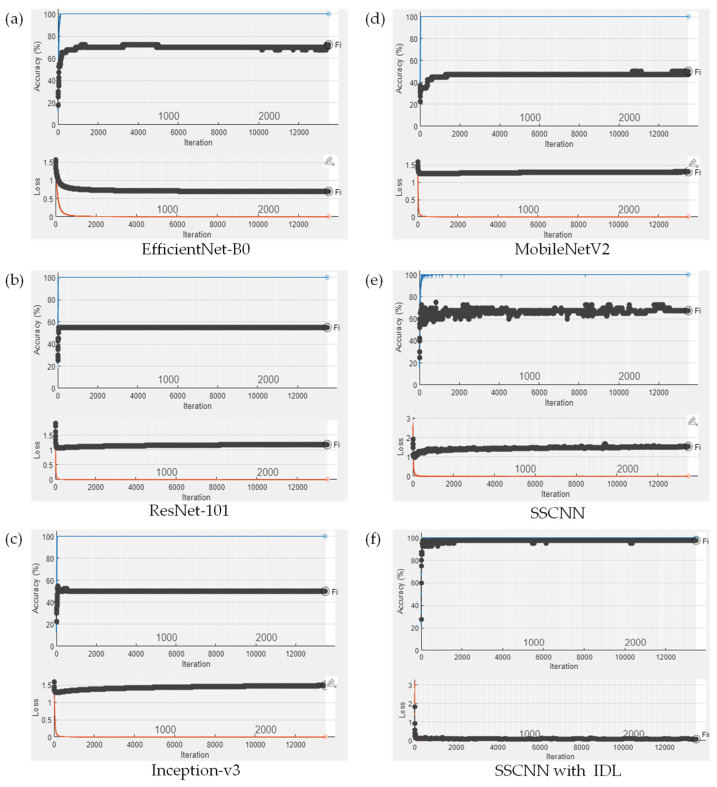
The training process on the SG3 testing dataset with and without the proposed IDL method. (**a**) EfficientNet-B0; (**b**) ResNet-101; (**c**) Inception-v3; (**d**) MobileNetV2; (**e**) SSCNN; (**f**) SSCNN with IDL. In the upper part of each figure, the blue line represents the training accuracy, and each epoch is denoted by a black dot indicating the recorded validation accuracy. In the lower part, the orange line represents the training loss, and each epoch corresponds to a black dot representing the recorded validation loss. It can be observed that all CNN models trained without the proposed IDL method (**a**–**e**) exhibited validation accuracies all below 75%, and all validation losses exceeded 0.5 throughout the training process. Notably, when combining the SSCNN with the proposed IDL method (**f**), after 1000 epochs, the validation loss consistently remained below 0.3, and the validation accuracy exceeded 95%. Moreover, the best validation accuracy achieved a satisfactory classification accuracy of 97.50%.

To validate the efficacy of the proposed IDL method, we created different state-of-the-art CNN models trained with the SG3 images before ROI cropping. The initial learning rate was set to 0.0001, and the batch size was set to 32. Stochastic gradient descent with momentum (SGDM) was employed as the optimization algorithm for all the training processes. To ensure a fair comparison, we fixed the training iteration at 13,500 for all the training processes. Figure 17 illustrates the training process of all the CNN models on the SG3 dataset, comparing the state-of-the-art CNN models trained without IDL and SSCNN with IDL. It can be observed that all the CNN models trained without IDL exhibited validation losses that kept exceeding 0.5, and the validation accuracies stayed below 75% throughout the training process.

In contrast, the proposed SSCNN with IDL consistently achieved validation losses below 0.3 and validation accuracies exceeding 95% after 1000 epochs. Notably, the best validation accuracy achieved a satisfactory classification accuracy of 97.50% in this training process. The comparison results are shown in Table 6. The results demonstrate that the proposed SSCNN with the IDL method achieves a 91.59% accuracy, much higher than other state-of-the-art CNN models. This shows that IDL can significantly improve the performance of CNN models for similar drug classifications within a similarity group.

### 3.4. Comparison of the IDL Effect for SG1-5

**Figure 18:**
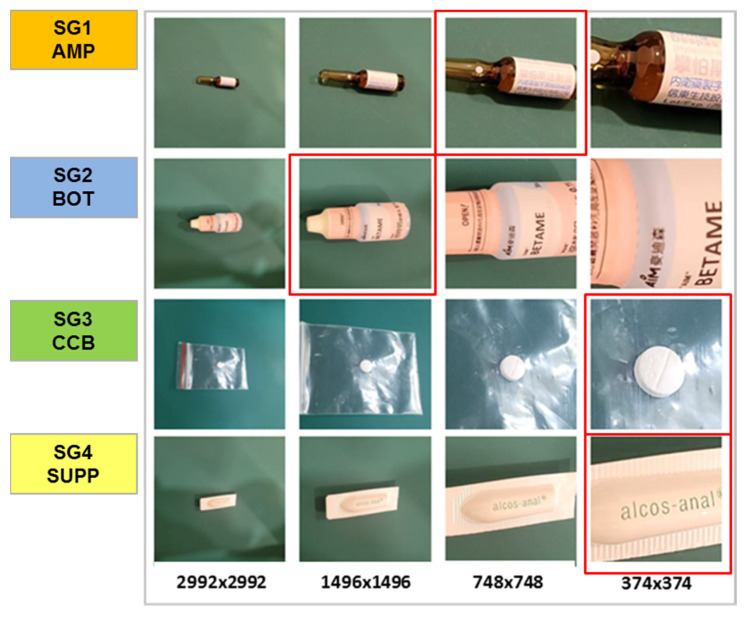
The cropping sizes and corresponding ROI images for SG1-4. In the AMP class, the pattern and color of the label are of paramount importance, leading us to select a size of 748 × 748. In the case of BOT, it is crucial to observe distinct bottle contours and color distribution, which led to the choice of 1496 × 1496. For CCB, a clear view of the tablet markings and labels is necessary. Thus, the size is set to 374 × 374. Regarding SUPP, capturing the distribution of label’s text is crucial. Hence, we opted for a 374 × 374 size.

In this section, the IDL effect for SG1-5 is discussed in detail. First, we applied the proposed IDL method to the SG1-4 images. We engaged a pharmacist with more than ten years of experience to evaluate the different drug features of SG1-4 and determine the optimal ROI for each SG. The ROI images were cropped for each SG, and the results were presented in Figure 18, followed by an evaluation and interpretation by the pharmacist. For AMP, contour features and proportions were deemed less significant, and the labels exhibited immense importance. In the case of BOT, both the contour details of the bottle and the differences in the labels needed to be considered, resulting in the preservation of the overall proportions and label distribution. For CCB, much of the packaging could be cropped, and we minimized the package reflection to enhance the clarity of the tablet imprints. For SUPP, detailed information about the label’s text and pattern distribution was deemed necessary, and thus we chose a larger cropping size. Considering the satisfactory classification performance of SG5 at its original size, we maintained the original size to reduce the labeling cost and computational burden associated with image-processing operations.

**Figure 19:**
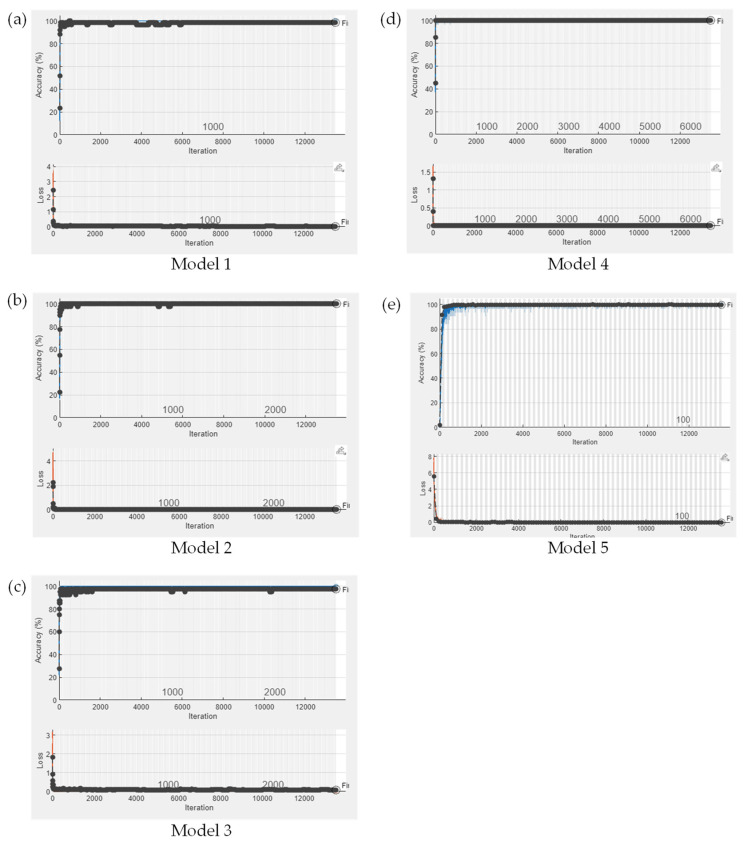
The training process for Models 1–5 was generated by SSCNN with IDL on SG1-5. (**a**) Model 1: AMP; (**b**) Model 2: BOT; (**c**) Model 3: CCB; (**d**) Model 4: SUPP; (**e**) Model 5: Other drugs. In the upper part of each figure, the blue line represents the training accuracy, and each epoch is denoted by a black dot indicating the recorded validation accuracy. In the lower part, the orange line represents the training loss, and each epoch corresponds to a black dot representing the recorded validation loss.

**Figure 20:**
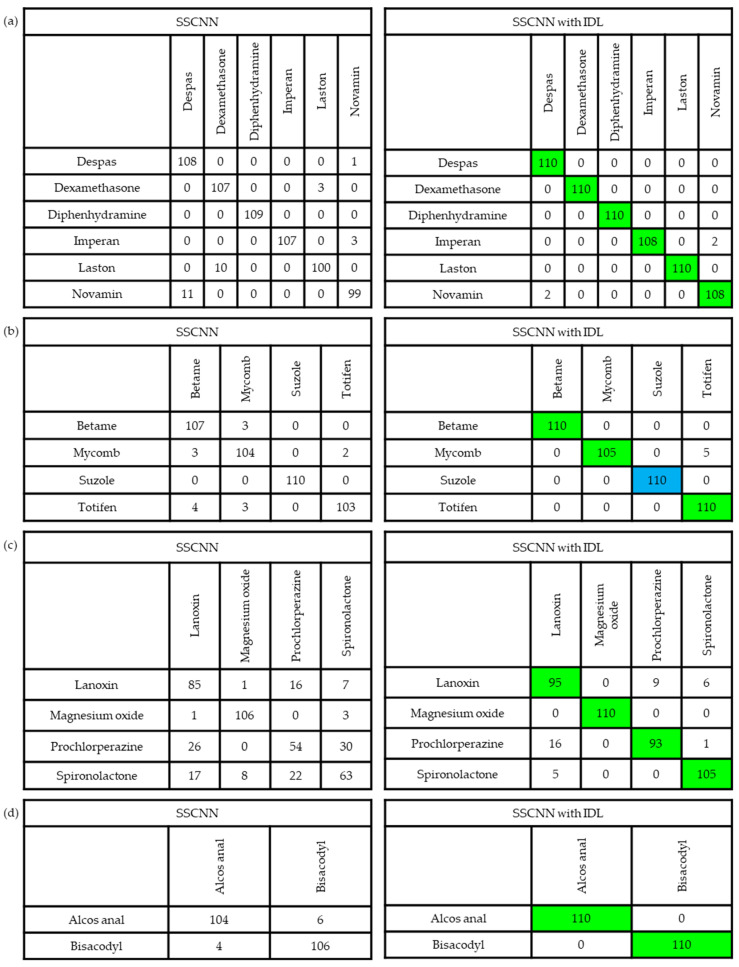
(**a**–**d**) are the 5-fold CVCM for SSCNN without and with IDL for SG1-4, respectively. The improved TP values are indicated in green, and the unchanged value is marked in blue. It can be observed that the IDL method resulted in significant improvements in the TP values of each SD across all SGs, except for Suzole, where the values remained unchanged. Particularly in SG3, there are significant improvements in TP values for all drugs. Prochlorperazine increased from 54 to 93, and Spironolactone increased from 63 to 105.

**Figure 21:**
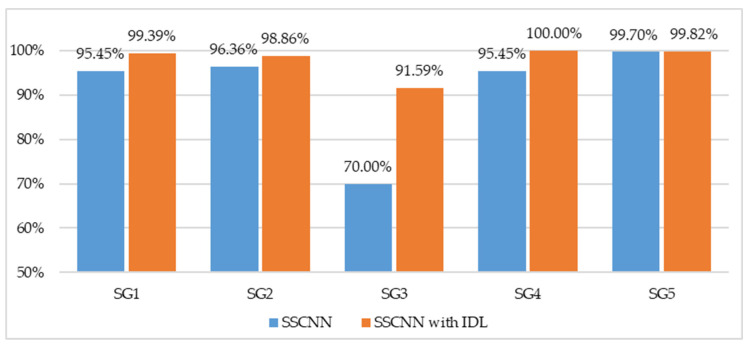
Testing accuracies for SG1-5 of SSCNN without and with IDL. The IDL improves accuracies for SG1-5. Specifically, there is a significant increase in accuracy for SG3 (CCB), which exhibited the lowest accuracy among all models. The accuracy significantly improved from 70.00% to 91.59%.

Once the pharmacist confirmed the selected optimal cropping sizes, we applied these sizes of images to train the proposed SSCNN; we defined this kind of training process as SSCNN with IDL. The training process is depicted in Figure 19, with Models 1–5 generated by SSCNN with IDL on SG1-5. During the training process, the validation loss dropped below 0.3 and the validation accuracy exceeded 98% after 6000 iterations for all the models. Furthermore, the best validation accuracies for Models 1–5 demonstrated their outstanding performance at 98.33%, 100%, 97.50%, 100.00%, and 99.89%, respectively. Furthermore, to verify whether each similar drug’s TP values improved after applying the proposed IDL method, we present the five-fold CVCM for each similar drug, shown in Figure 20. The five-fold CVCM was used to evaluate the improvement in the TP values for each similar drug. Observing the five-fold CVCMs for each similar drug shows that the TP values for each group showed improvement after applying the proposed IDL. Figure 21 depicts the testing accuracy improvement for Models 1–5. Specifically, the testing accuracy of Model 3 significantly improved from 70.00% to 91.59%, and that of Model 4 improved from 95.45% to 100%. This improvement indicates that the proposed IDL effectively enhances the drug-classification accuracy for Models 1–5, particularly for Model 3, where significant enhancements are achieved. It suggests that the proposed IDL method significantly improves the accuracy of Models 1–5, enabling a more precise differentiation of similar drugs.

### 3.5. Results of the TSIDL

This section provides a detailed discussion of the testing results by combining a two-stage CNN framework with the proposed IDL, which is the proposed TSIDL method. We applied the two-stage inference framework proposed in Section 2.2.4 to evaluate the proposed TSIDL method. The evaluations for the proposed TSIDL method were performed on a 13th generation Intel(R) Core (TM) i5-13500 processor with 64 GB of memory and an NVIDIA GeForce RTX 3060 GPU. In the first stage, Model 0 generated the first inference results at the drug-name level and package level. Subsequently, we compared these inference results for drug names with the SG map and assigned them to the corresponding SGs. Then, the SG1-5 images were cropped by using their respective optimal ROI images. In the second stage, Models 1–5 were employed to classify these optimal ROI images and generate the final inference results. Finally, the inference results from Models 1–5 were aggregated to obtain the overall inference results of TSIDL.

We compared the proposed TSIDL method with other state-of-the-art CNN models, and the results are shown in Table 7. For a fair comparison, all the models were trained for 100 epochs (13,500 iterations). The results show that the proposed TSIDL method outperformed the other models in all classification metrics. It achieved a recall of 99.39%, precision of 99.41%, F1 score of 99.39%, and accuracy of 99.39%. The inference time per image of TSIDL is only 3.12 ms. We further compare the experimental results with other state-of-the-art CNN models. Regarding the classification accuracy, EffcientNet-B0 achieves only the second-highest classification accuracy of 99.18%, while TSIDL achieves the highest classification accuracy of 99.39%. TSIDL outperforms EffcientNet-B0 in terms of classification accuracy. Regarding the inference time, SSCNN achieves the fastest inference speed of 1.17 ms. Although TSIDL ranked fourth in inference time per image, it is still superior to larger models such as ResNet-101 and Inception-v3. Importantly, in the applications of drug classification, accurate classification results are important for patient medication safety. This research achieved a state-of-the-art accuracy, demonstrating the effectiveness of the proposed TSIDL method in significantly improving the diverse-packaging-drug-classification performance.

## 4. Discussion

To our knowledge, this study represents the first attempt to apply deep learning to classify drugs with diverse packaging. Compared to classifying drugs with the same type of packaging, this is more relevant to a pharmacy’s operational scenarios. Additionally, we discovered that some similar drugs among the drugs with diverse packaging led to higher classification errors than other drugs. Therefore, we proposed a novelty grouping algorithm to group these similar drugs to the similarity groups. Considering each similarity group’s drug characteristics, we propose a novel IDL method to enhance the classification performance of each similarity group. By selecting the optimal ROIs for each similarity group for model training, the classification performance achieved significant improvements in all similarity groups, particularly for classifying CCB-packaged pills. The classification accuracy significantly improved from 70.00% to 91.59%.

We further combine the two-stage CNN framework with the proposed IDL, which is the proposed TSIDL method. The experimental results demonstrate that the proposed TSIDL method outperforms other state-of-the-art models in all classification metrics. It achieves a recall of 99.39%, a precision of 99.41, an F1 score of 99.39%, and an accuracy of 99.39%. It is worth noting that in the actual dispensing process, accurate classification results are crucial for patient medication safety. Additionally, the inference time per image of TSIDL is only 3.12 ms, which shows that TSIDL has the potential for real-time clinical applications. Moreover, this study achieves a state-of-the-art accuracy, demonstrating the effectiveness of the proposed TSIDL method in diverse-packaging drug classification. These results highlight the potential for the real-time classification of similar drugs with diverse packaging and their applications in future dispensing systems, which can prevent dispensing errors from occurring and ensure patient safety efficiently.

Due to time constraints in data collection, we utilized a dataset of 108 common diverse-packaging drug pictures as proof of concept. Considering that real medical institutions typically stock medicines with thousands of package types, our future work will add more pictures of various packaging types, which can expand the dataset to include a broader range of drug classes and packaging types to investigate the performance of deep learning models across a broader spectrum of diverse-packaging drugs.

Moreover, in clinical practice, there can be instances where newly introduced drugs are not included in our training set. Although the proposed TSIDL model has potential for clinical applications, there exists a risk of misclassifying the newly introduced drugs before the model’s training is complete. Future work can consider incorporating unsupervised-learning-based clustering before the input layer of TSIDL, which can exclude untrained drug items and prevent our model from erroneously classifying the newly introduced drugs.

## 5. Conclusions

In this study, we constructed a dataset of 108 drugs distributed across 12 package types, comprising 7776 images. This dataset differed from previous databases that focused on drugs with the same type of packaging. We applied the proposed SSCNN and achieved a drug-classification accuracy of 98.16%. Furthermore, we investigated similar drugs among the dataset and proposed an efficient grouping algorithm, achieving a group-level classification accuracy of 99.92%. Additionally, we determined the optimal ROIs for the five similarity groups (SGs) and propose a novel IDL method. The IDL method improved the accuracy for all five SGs, particularly SG3, which significantly increased from 70.00% to 91.59%. Further, we propose a novel TSIDL method that outperforms other state-of-the-art CNN models in all classification metrics. It achieved a state-of-the-art classification accuracy of 99.39%. Additionally, the inference time per image of TSIDL is only 3.12 ms. These results highlight the potential of real-time classification for similar drugs with diverse packaging and their applications in future dispensing systems. By applying the proposed TSIDL model with a general mobile phone camera, it can be easily integrated with existing dispensing systems to more accurately categorize medications, preventing dispensing errors and ensuring patient safety efficiently.

## Figures and Tables

**Table 1:** The medication list in this study. The list includes 108 medications distributed among 12 different types of packaging.

Package Type	Drug Name	Package Type	Drug Name
Amp	Despas	Syringe	Clexane
Amp	Dexamethasone	Syringe	Humalog
Amp	Diphenhydramine	Syringe	Levemir
Amp	Furosemide	Syringe	NESP
Amp	Imperan	Syringe	Novomix
Amp	Laston	Syringe	Novorapid
Amp	Novamin	Syringe	Recormon 2000
Amp	Tranexamic acid	Syringe	Recormon 5000
Amp	Voren	Syringe	Toujeo
Blister	Anpo	Syrup	Anti-Phen
Blister	Anwu	Syrup	Aswell
Blister	Bafen	Syrup	Brown mixture
Blister	Dogmatyl	Syrup	Cetirizine
Blister	Dorison	Syrup	Cypromin
Blister	Euricon	Syrup	Kidsolone
Blister	Nebilet	Syrup	Meptin
Blister	Syntam	Syrup	Musco
Blister	Terodine	Syrup	Secorine
Bot	Alminto	Tube	Biomycin
Bot	Altropine0-125	Tube	Fusidic acid
Bot	Altropine0-3	Tube	Gentaderm
Bot	Betame	Tube	Gentamicin
Bot	Exelderm	Tube	Mycomb
Bot	Eyehelp	Tube	Nincort
Bot	Flucason	Tube	Sinpharderm
Bot	Jaline	Tube	Tetracycline
Bot	Mycomb	Tube	Topsym
Bot	Suzole	Vial	Cefa
Bot	Topsym	Vial	Gentamycin
Bot	Totifen	Vial	Lofatin
ClipChainBag	Ceficin	Vial	Medason
ClipChainBag	Erythromycin	Vial	Mepem
ClipChainBag	Kentamin	Vial	Sintrix
ClipChainBag	Lanoxin	Vial	Soonmelt
ClipChainBag	Ligilin	Vial	Subacillin
ClipChainBag	Magnesium oxide	Vial	U-Vanco
ClipChainBag	Prochlorperazine	PaperBox	Azetin
ClipChainBag	Spironolactone	PaperBox	Berotec
ClipChainBag	Transamin	PaperBox	Ciproxin
Enema	Sumgel	PaperBox	Claforan
Powder	Actein	PaperBox	Cravit
Powder	Biofermin R	PaperBox	Depakine
Powder	Forlax	PaperBox	Invanz
Powder	Hidrasec infant	PaperBox	Nimotop
Powder	Histapp	PaperBox	Relvar
Powder	Kalimate	PaperBox	Solu
Powder	Miyarisan	PaperBox	Spiolto
Powder	Normacol Plus	PaperBox	Spiriva
Powder	Smecta	PaperBox	Stilamin
Supp	Albothyl	PaperBox	Symbicort rapihaler
Supp	Alcos anal	PaperBox	Symbicort turbuhaler
Supp	Bisacodyl	PaperBox	Trelegy
Supp	Frotin	PaperBox	Trisonin
Supp	Voren	PaperBox	Vfend

**Table 2:** Proposed SSCNN based on AlexNet. The SSCNN architecture consists of eight layers, including five convolutional layers followed by three fully connected layers. We adjusted the output layer of AlexNet to accommodate the classification of 108 different packaging medications in this study.

Layer	Type	Maps	Size	Kernel Size	Stride	Padding	Activation
Input	Input Layer	3	227 × 227	-	-	-	-
Conv1	Convolution	96	55 × 55	11 × 11	4	0	ReLU
Pool1	Max Pooling	96	27 × 27	3 × 3	2	0	-
Conv2	Convolution	256	27 × 27	5 × 5	1	2	ReLU
Pool2	Max Pooling	256	13 × 13	3 × 3	2	0	-
Conv3	Convolution	384	13 × 13	3 × 3	1	1	ReLU
Conv4	Convolution	384	13 × 13	3 × 3	1	1	ReLU
Conv5	Convolution	256	13 × 13	3 × 3	1	1	ReLU
Pool5	Max Pooling	256	6 × 6	3 × 3	2	0	-
Fc6	Fully Connected	-	4096 × 1	-	-	-	ReLU
Fc7	Fully Connected	-	4096 × 1	-	-	-	ReLU
Fc8	Fully Connected	-	108 × 1	-	-	-	Softmax

**Table 3:** The drug- and package-level classification results of the first-stage deep learning model.

Classify Target	Recall	Precision	F1-Score	Accuracy
Drug Name	98.16%	98.19%	98.13%	98.16%
Package	99.97%	99.98%	99.97%	99.97%

**Table 4:** Examples of similar drugs for SG1-4.

Similar Group	Drugs of Similarity Group					
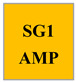	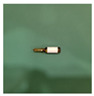	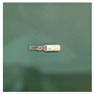	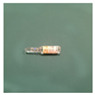	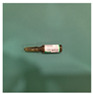	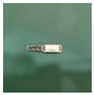	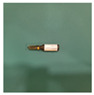
	Despas	Dexamethasone	Diphenhydramine	Imperan	Laston	Novamin
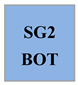	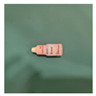	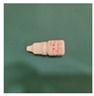	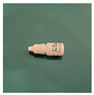	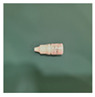		
	Betame	Mycomb	Suzole	Totifen		
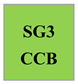	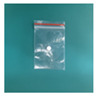	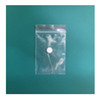	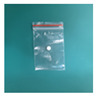	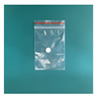		
	Lanoxin	Lanoxin	Prochlorperazine	Spironolactone		
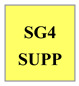	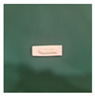	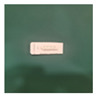				
	Alcos anal	Bisacodyl				

**Table 5:** The grouping level classification results achieved superior accuracy. Only a few drugs were misclassified.

Classify Target	Recall	Precision	F1-Score	Accuracy
Similarity Group	99.84%	99.65%	99.74%	99.92%

**Table 6:** A comparison between the proposed SSCNN with the IDL and the state-of-the-art CNN models without the IDL on the SG3 testing dataset. The proposed SSCNN with the IDL achieves a 91.59% testing accuracy, much higher than other state-of-the-art CNN models. Bold indicates the best performance.

Model	Recall	Precision	F1-Score	Accuracy
EfficientNet-B0 [34]	59.55%	60.37%	59.13%	59.55%
ResNet-101 [35]	61.14%	61.59%	61.06%	61.14%
Inception-v3 [36]	53.64%	53.71%	53.37%	53.64%
MobileNetV2 [37]	52.27%	53.55%	52.57%	52.27%
SSCNN	66.36%	67.55%	66.49%	66.36%
SSCNN with IDL	**91.59%**	**91.77%**	**91.58%**	**91.59%**

**Table 7:** Comparison of the overall performance between the proposed TSIDL and the state-of-the-art CNN models. Bold indicates the best performance.

Model	Recall	Precision	F1-Score	Accuracy	Inference Time (ms)
EfficientNet-B0 [34]	99.18%	99.22%	99.18%	99.18%	2.54
ResNet-101 [35]	98.69%	98.77%	98.69%	98.69%	3.26
Inception-v3 [36]	98.46%	98.54%	98.43%	98.46%	3.40
MobileNetV2 [37]	98.79%	98.84%	98.78%	98.79%	1.54
SSCNN	98.16%	98.19%	98.13%	98.16%	**1.17**
Proposed TSIDL	**99.39%**	**99.41%**	**99.39%**	**99.39%**	3.12

## Data Availability

Data are available upon request.

## References

[1] Makary M.A., Daniel M. (2016). Medical error—The third leading cause of death in the US. Br. Med. J..

[2] James K.L., Barlow D., Burfield R., Hiom S., Roberts D., Whittlesea C. (2011). Unprevented or prevented dispensing incidents: Which outcome to use in dispensing error research?. Int. J. Pharm. Pract..

[3] World Health Organization Home Page. https://www.who.int/initiatives/medication-without-harm.

[4] Greene J.A., Kesselheim A.S. (2011). Why do the same drugs look different? Pills, trade dress, and public health. N. Engl. J. Med..

[5] Ostini R., Roughead E.E., Kirkpatrick C.M., Monteith G.R., Tett S.E. (2012). Quality Use of Medicines-medication safety issues in naming; look-alike, sound-alike medicine names. Int. J. Pharm. Pract..

[6] Ciapponi A., Fernandez Nievas S.E., Seijo M., Rodríguez M.B., Vietto V., García-Perdomo H.A., Virgilio S., Fajreldines A.V., Tost J., Rose C.J. (2021). Reducing medication errors for adults in hospital settings. Cochrane Database Syst. Rev..

[7] Cheung K.C., Bouvy M.L., De Smet P.A. (2009). Medication errors: The importance of safe dispensing. Br. J. Clin. Pharmacol..

[8] Aldhwaihi K., Schifano F., Pezzolesi C., Umaru N. (2016). A systematic review of the nature of dispensing errors in hospital pharmacies. Integr. Pharm. Res. Pract..

[9] Larmené-Beld K.H.M., Alting E.K., Taxis K. (2018). A systematic literature review on strategies to avoid look-alike errors of labels. Eur. J. Clin. Pharmacol..

[10] Takase T., Masumoto N., Shibatani N., Matsuoka Y., Tanaka F., Hirabatake M., Kashiwagi H., Nishioka I., Ikesue H., Hashida T. (2022). Evaluating the safety and efficiency of robotic dispensing systems. J. Pharm. Health Care Sci..

[11] Ahtiainen H.K., Kallio M.M., Airaksinen M., Holmström A.R. (2020). Safety, time and cost evaluation of automated and semi-automated drug distribution systems in hospitals: A systematic review. Eur. J. Hosp. Pharm..

[12] Berdot S., Boussadi A., Vilfaillot A., Depoisson M., Guihaire C., Durieux P., Le L.M.M., Sabatier B. (2019). Integration of a Commercial Barcode-Assisted Medication Dispensing System in a Teaching Hospital. Appl. Clin. Inform..

[13] Yaniv Z., Faruque J., Howe S., Dunn K., Sharlip D., Bond A., Perillan P., Bodenreider O., Ackerman M.J., Yoo T.S. The National Library of Medicine Pill Image Recognition Challenge: An Initial Report. Proceedings of the 2016 IEEE Applied Imagery Pattern Recognition Workshop (AIPR).

[14] Zeng X., Cao K., Zhang M. MobileDeepPill: A small-footprint mobile Deep learning system for recognizing unconstrained pill images. Proceedings of the 15th Annual International Conference on Mobile Systems, Applications, and Services.

[15] Usuyama N., Delgado N.L., Hall A.K., Lundin J. ePillID Dataset: A Low-Shot Fine-Grained Benchmark for Pill Classification. Proceedings of the 2020 IEEE/CVF Conference on Computer Vision and Pattern Recognition Workshops (CVPRW).

[16] Ling S., Pastor A., Li J., Che Z., Wang J., Kim J., Le Callet P. Few-Shot Pill Recognition. Proceedings of the 2020 IEEE/CVF Conference on Computer Vision and Pattern Recognition (CVPR).

[17] Kwon H.J., Kim H.G., Lee S.H. (2022). Pill Detection Model for Medicine Inspection Based on Deep Learning. Chemosensors.

[18] Patel U. Machine Learning-based Pharmaceutical Tablet Inspection and Recognition Techniques—A Review. Proceedings of the 2021 International Conference on Artificial Intelligence and Smart Systems (ICAIS).

[19] Cha K., Woo H.K., Park D., Chang D.K., Kang M. (2021). Effects of Background Colors, Flashes, and Exposure Values on the Accuracy of a Smartphone-Based Pill Recognition System Using a Deep Convolutional Neural Network: Deep Learning and Experimental Approach. JMIR Med. Inform..

[20] Zheng A., Yang H., Pan X., Yin L., Feng Y. (2021). Identification of Multi-Class Drugs Based on Near Infrared Spectroscopy and Bidirectional Generative Adversarial Networks. Sensors.

[21] Yang Z., Bai J. Vial bottle mouth defect detection based on machine vision. Proceedings of the 2015 IEEE International Conference on Information and Automation.

[22] Rawashdeh N.A., Abu-Khalaf J.M., Khraisat W., Al-Hourani S.S. (2018). A visual inspection system of glass ampoule packaging defects: Effect of lighting configurations. Int. J. Comput. Integr. Manuf..

[23] Liu X., Meehan J., Tong W., Wu L., Xu X., Xu J. (2020). DLI-IT: A deep learning approach to drug label identification through image and text embedding. BMC Med. Inform. Decis. Mak..

[24] Gromova K., Elangovan V. (2022). Automatic Extraction of Medication Information from Cylindrically Distorted Pill Bottle Labels. Mach. Learn. Knowl. Extr..

[25] Wang J.S., Ambikapathi A., Han Y., Chung S.L., Ting H.W., Chen C.F. Highlighted Deep Learning based Classification of Pharmaceutical Blister Packages. Proceedings of the 2018 IEEE 23rd International Conference on Emerging Technologies and Factory Automation (ETFA).

[26] Han Y., Chung S.L., Xiao Q., Wang J.S., Su S.F. (2021). Pharmaceutical Blister Package Classification Based on Induced deep learning. IEEE Access.

[27] Ting H.W., Chung S.L., Chen C.F., Chiu H.Y., Hsieh Y.W. (2020). A drug classification model developed using deep learning technologies: Experience of a medical center in Taiwan. BMC Health Serv. Res..

[28] Dey M., Mia S.M., Sarkar N., Bhattacharaya A., Roy S., Malakar S., Sarkar R. (2021). A two-stage CNN-based hand-drawn electrical and electronic circuit component recognition system. Neural Comput. Appl..

[29] Bianco S., Cadene R., Celona L., Napoletano P. (2018). Benchmark Analysis of Representative Deep Neural Network Architectures. IEEE Access.

[30] Krizhevsky A., Sutskever I., Hinton G.E. (2012). ImageNet Classification with Deep Convolutional Neural Networks. Advances in Neural Information Processing Systems, Proceedings of the 26th Annual Conference on Neural Information Processing Systems 2012, Lake Tahoe, NV, USA, 3–6 December 2012.

[31] Germain Lee B.C. (2019). Machine learning, template matching, and the International Tracing Service digital archive: Automating the retrieval of death certificate reference cards from 40 million document scans. Digit. Scholarsh. Humanit..

[32] The MathWorks Inc. (2023). MATLAB Version: 9.14.0 (R2023a), Natick, Massachusetts: The MathWorks Inc. https://www.mathworks.com.

[33] Takahashi K., Yamamoto K., Kuchiba A., Koyama T. (2022). Confidence interval for micro-averaged F1 and macro-averaged F1 scores. Appl. Intell..

[34] Tan M., Le Q.V. (2019). EfficientNet: Rethinking Model Scaling for Convolutional Neural Networks. arXiv.

[35] He K., Zhang X., Ren S., Sun J. Deep residual learning for image recognition. Proceedings of the IEEE conference on computer vision and pattern recognition.

[36] Szegedy C., Vanhoucke V., Ioffe S., Shlens J., Wojna Z. Rethinking the Inception Architecture for Computer Vision. Proceedings of the 2016 IEEE Conference on Computer Vision and Pattern Recognition (CVPR).

[37] Sandler M., Howard A., Zhu M., Zhmoginov A., Chen L.C. MobileNetV2: Inverted Residuals and Linear Bottlenecks. Proceedings of the IEEE/CVF Conference on Computer Vision and Pattern Recognition.

